# The Management of an Open Biopsy Tract Site Infection and Ulceration in the Setting of Osteosarcoma

**DOI:** 10.5435/JAAOSGlobal-D-22-00038

**Published:** 2022-07-06

**Authors:** Stephen Bowen, Eric Lepkowsky, Fazel Khan

**Affiliations:** From the Department of Orthopaedics, Stony Brook Medicine, Stony Brook, NY.

## Abstract

Biopsy site infection in the setting of osteosarcoma is a potentially devastating complication. We present the case of a 16-year-old adolescent girl with a distal femur osteosarcoma who developed an open biopsy site ulceration and infection after initiation of neoadjuvant chemotherapy. This was treated with careful local excision of the ulcerated biopsy site and systemic antibiotic therapy throughout the duration of her chemotherapy course. She subsequently underwent wide resection of the tumor en bloc with a generous ellipse around the biopsy scar and reconstruction with cemented knee megaprosthesis. No recurrence of either infection or malignancy was observed. This case represents the successful treatment of a biopsy site ulceration and infection in a patient with osteosarcoma and may merit adoption in future instances of this complication.

Osteosarcomas are aggressive primary bone tumors, representing approximately 2% of all pediatric cancers.^1^ Advancements in medical and surgical management have drastically improved survival and enabled limb-sparing surgery (LSS) in most cases.^2,3^ Prompt diagnosis and treatment is necessary to take advantage of these more updated strategies.

Definitive diagnosis by biopsy is required before initiating treatment.^4^ Complications after biopsy are rare and usually minor; however, some biopsy complications have devastating sequelae. For example, inappropriately performed biopsies can cause undesired extensive local tumor contamination, potentially compromising limb salvage options.^5^ In addition, a biopsy site infection or any infection during chemotherapy can be catastrophic, increasing the likelihood of amputation over limb salvage and decreasing survival because of chemotherapy delays.^4,6,7^ Thus, it is critical to treat any infection quickly and aggressively.

There is a paucity of information regarding infected biopsy sites and their management in the setting of osteosarcoma. We present a case of a 16-year-old adolescent girl with distal femur osteosarcoma who developed a biopsy site infection and was successfully treated with no long-term complications and no recurrence of either infection or osteosarcoma.

## Case Presentation

**Figure 1:**
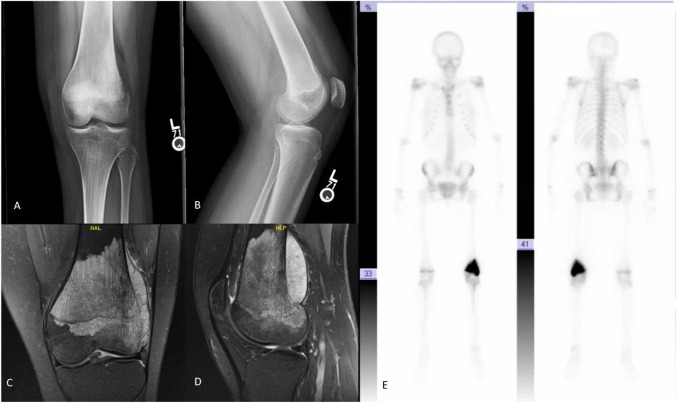
**A**, AP radiograph of the patient's left knee. **B**, Lateral radiograph of the patient's left knee. **C**, Coronal MRI cuts of the patient's left knee. **D**, Sagittal MRI cuts of the patient's left knee. **E**, Whole body bone scan of the patient with increased uptake in the left knee.

The patient is a 16-year-old adolescent girl who developed left knee pain after falling off a trampoline. A radiograph of the left knee demonstrated a destructive lesion within the distal femur (Figure 1). Chest CT, bone, and MRI scans of the femur were obtained (Figure 1). MRI and bone scans demonstrated a distal femoral lesion concerning for malignancy. Chest CT revealed multiple nodules throughout bilateral lungs, which was concerning for metastases.

**Figure 2:**
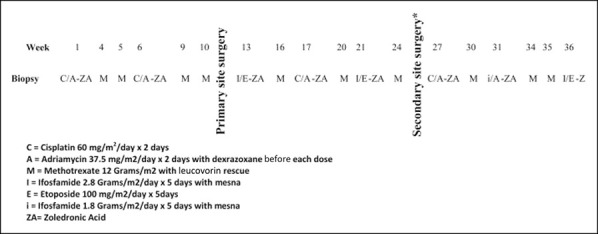
Diagram showing the original chemotherapy protocol AOST06P1.

She underwent open biopsy of the femur lesion the week of initial presentation. The biopsy was done through a longitudinal (lateral) incision, and strict biopsy principles were adhered to. Cefazolin was given after intraoperative cultures were obtained. Pathologic analysis confirmed diagnosis of chondroblastic osteosarcoma, and the patient was initiated on chemotherapy protocol AOST06P1 (Figure 2) on postoperative day 15.^8^ The patient was seen 10 days after surgery, and the biopsy site was found to be healing without any complications. She was cleared for initiation of chemotherapy the following week.

During week 2 of chemotherapy, erythema was noted around the biopsy site with a 1.5-cm ulcer at its distal aspect. The patient was found to be febrile to 38.2°C with an absolute neutrophil count of 45 cells/μL. Vancomycin and cefepime were initiated, and chemotherapy was paused to allow the absolute neutrophil count to normalize. Owing to persistent erythema and ulceration, the patient underwent surgery to address the infected biopsy site. We used an ellipse of the ulcerated area, extending approximately 10 mm away from the incision line (3 × 2 cm) where healthy skin tissue was noted. Cultures were obtained. Gentle irrigation was done with a handheld bulb syringe, and great care was taken to avoid undermining the surrounding tissue. Meticulous hemostasis was again obtained. A negative pressure wound therapy (NPWT) sponge was placed. The wound cultures grew pan-sensitive *Pseudomonas aeruginosa*. She returned to the operating room 2 days later for staged closure with the plastic surgery team. The closure was done in a layered fashion: 3-0 interrupted braided suture for the fascia, 3-0 interrupted monofilament for the dermis, and a 3-0 running subcuticular monocryl layer. A drain was placed at this time longitudinally in line with the incision. This was removed 2 days later. On discharge, she was transitioned from intravenous antibiotics to oral sulfamethoxazole-trimethoprim, which was intermittently replaced with intravenous cefepime during methotrexate administrations to avoid folate depletion.

**Figure 3:**
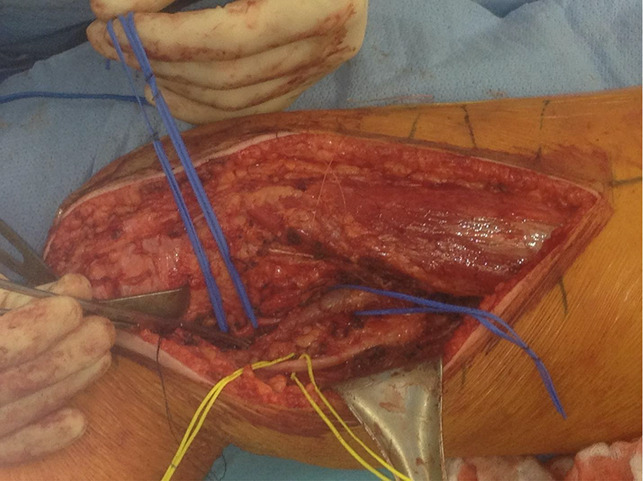
Photograph showing the exposure with preservation of neurovascular structures

**Figure 4:**
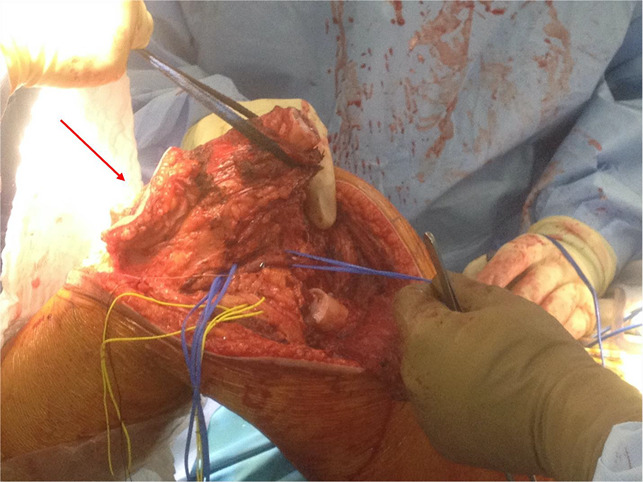
Photograph showing wide resection of the tumor with the arrow denoting large ellipse around the formerly infected biopsy site

**Figure 5:**
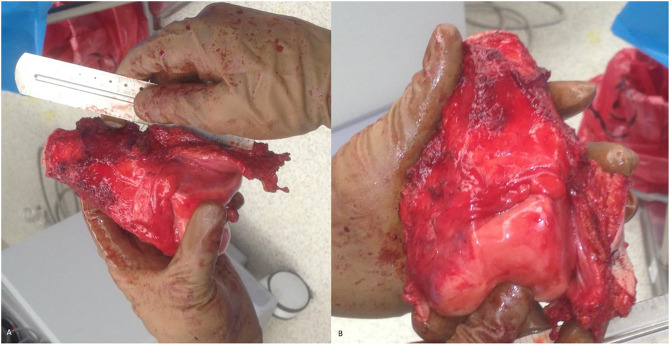
Photographs showing a resected tumor with surrounding cuff of normal tissue

**Figure 6:**
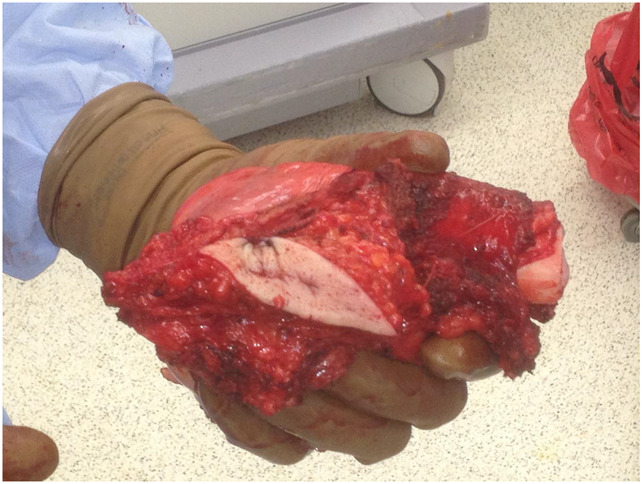
Photograph showing that wide ellipse was used to excise the site of the infected biopsy tract.

This regimen continued until planned wide resection of the tumor, which included the infected biopsy tract. An exceptionally wide ellipse was used to excise the biopsy tract and drain the site, given the history of infection and repeat surgery with possible contamination. This extended several centimeters in all directions and was beveled away from the site to ensure wide margins were obtained. Wide resection of the tumor was then conducted, with care taken to protect the surrounding neurovascular structures (Figures 3–6).

**Figure 7:**
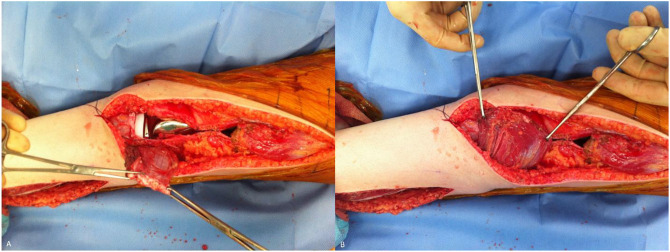
Photographs showing that a lateral gastrocnemius flap was used by the plastic surgery team for soft-tissue coverage.

Reconstruction was done with a cemented knee megaprosthesis, and the plastic surgery team used a gastrocnemius flap to provide soft-tissue coverage (Figure 7). A drain was placed, and intravenous cefazolin was continued until drain removal. Afterward, the patient received oral cefazolin until discharge, at which time the sulfamethoxazole-trimethoprim regimen was resumed. She was maintained in a knee immobilizer postoperatively to facilitate wound healing.

The patient continued with scheduled chemotherapy after resection. There were multiple episodes of neutropenic fever throughout her course during which times sulfamethoxazole-trimethoprim was suspended and replaced with intravenous cefepime, nafcillin, and vancomycin. Blood cultures were negative in all instances. Sulfamethoxazole-trimethoprim was stopped in week 17 because of concerns for allergic reaction causing a rash, and monthly pentamidine infusions were initiated instead. The patient had one additional episode of febrile neutropenia 4 months later with blood cultures positive for *Klebsiella pneumonia*. This was successfully treated with oral ciprofloxacin. She had no subsequent occurrences of febrile neutropenia or presumed sepsis.

The complete antimicrobial course included systemic antibiotics for a total of 41 weeks. She has had no additional concerns for infection. The only additional complication was stiffness of her surgical knee, which required manipulation under anesthesia. Her most recent follow-up, 9 years since resection, demonstrated no evidence of disease recurrence and no complications with her reconstruction.

## Discussion

Osteosarcomas are aggressive primary bone tumors, representing 1.5% to 2.5% of all pediatric cancers.^1^ Historical survival was poor, ranging from 10% to 22% at 5 years.^9^ Treatment originally consisted of amputation, which was often ineffective because 80% of cases had lung metastasis on presentation.^10^ Contemporary treatment of osteosarcoma now consists of neoadjuvant chemotherapy, followed by wide surgical resection and adjuvant chemotherapy.^9^ This has allowed for LSS with 5-year survival rates of up to 70%.^9^

A biopsy must be obtained before the initiation of treatment. CT-guided biopsy has an accuracy ranging from 66% to 98%; however, open incisional biopsy remains the benchmark with an accuracy from 91% to 96%.^11^ Overall complication rates due to biopsy vary, approximately 1% in CT-guided core biopsy and as high as 16% in open biopsy.^12^ Common complications include infection, seroma, dehiscence, and hematoma, each of which may predispose for local tumor recurrence.^4,13,14^

Infection is particularly concerning in these patients given their susceptibility and the grave consequences. Intensive chemotherapy, long-term central venous catheters, and large resection voids increase infection risk.^1,6^ A concern in these patients is that chemotherapy must often be suspended to successfully clear the infection.^7^ This can negatively affect overall survival and increase the necessity of amputation rather than LSS.^1,7^

Biopsy tract or surgical site infections create a more complicated tumor bed and increase the risk of local contamination by spread through surrounding cellulitis or edema and by the tumorigenic effect of many inflammatory markers.^5,15^ Because of these risks in our patient, the ulcerated and infected tissue was thoroughly excised. In addition, at the time of wide resection, a wider than normal ellipse around the biopsy site was used to remove any potentially contaminated tissue. During the initial excision of infected tissue, great care was taken to avoid undermining the surrounding area because elevating large flaps or crossing tissue planes is well known to increase the risk of tumor contamination.^16,17^

The use of NPWT in the oncologic setting is controversial. Historically, malignancy has been considered a contraindication to NPWT because it was thought to stimulate tumor growth.^18^ However, more recent studies have supported its use, reporting no difference in recurrence-free survival and markedly decreased infection rates when NPWT is used.^19,20^ Given the concern for infection in our case and the necessity for timely and effective healing, the decision was made to use a wound VAC.

A large amount of resection void in these cases is often unavoidable because of the magnitude of tissue removal required.^21^ Primary closure without addressing this can result in hematoma formation with resultant infection or necrosis of the overlying soft tissue.^22^ Addressing this space is thus essential to decrease infection risk, provide adequate coverage of the prosthesis, and support the vascularity of the overlying skin.^23^ The decision was made in our case to proceed with aggressive soft-tissue coverage, and lateral gastrocnemius flap transposition was done at the time of reconstruction.

Despite the attempts taken to control the infection in this case, a low threshold was maintained to proceed with amputation, if necessary, to allow resumption of chemotherapy. As stated earlier, it is crucial to minimize chemotherapy delays to increase overall survival.^1,7^ It is possible that the infection did result in a degree of local contamination that escaped the initial excision and later wide resection. In this case, our success would likely have been aided by the chemotherapy's effect on residual tumor. However, interestingly, the necrosis rate of the patient's tumor was ultimately found to be 58%, indicating a poor response to the therapy.^24^

Reconstruction options after distal femur osteosarcoma resection include femoral rotationplasty, allograft, distraction osteogenesis, and megaprosthetic reconstruction.^25^ After presenting these options to the patient and family, they chose to proceed with megaprosthesis reconstruction. This modality is often chosen because it provides rapid restoration of function and is associated with good long-term outcomes. Unfortunately, deep infection occurs at a higher incidence than with primary arthroplasty (8% to 15% versus 1% to 2%).^26^ Local treatment of periprosthetic infection is rarely successful, and revision surgery combined with extended systemic antibiotic therapy is often required.^27^ This again reinforces the concept that it is critical to conduct all possible measures to minimize the chances of deep infection.

## Conclusion

Biopsy site infection in the setting of osteosarcoma is a challenging problem with limited treatment recommendations examined in the literature. In our case, a 16-year-old patient with a biopsy site infection was successfully treated with a combination of local excision of the infected area and antibiotic therapy throughout the duration of her chemotherapy course. This resulted in eradication of the infection and a relatively uncomplicated course. We think that for osteosarcoma, continuing antibiotics for the duration of chemotherapy may represent an approach to biopsy site infections that may minimize the risk of deep periprosthetic infection. Additional investigation into this protocol and antibiotic choice is warranted.
